# Hepatic Actinomycosis Mimicking Malignancy After Pancreaticoduodenectomy: A Case Report

**DOI:** 10.7759/cureus.21234

**Published:** 2022-01-14

**Authors:** Luisa Frutuoso, Ana Marta Pereira, Vera Oliveira, Gil Gonçalves, Mário Nora

**Affiliations:** 1 General Surgery, Centro Hospitalar de Entre Douro e Vouga, Santa Maria da Feira, PRT; 2 General Surgery, Champalimaud Foundation, Lisbon, PRT; 3 General Surgery, Centro Hospitalar Entre o Douro e Vouga, Santa Maria da Feira, PRT

**Keywords:** liver actinomycosis, hepatic pseudotumor, granulomatous disease, hepatic neoplasm, actinomyces infection

## Abstract

Abdominal actinomycosis is a rare disease caused by a Gram-positive bacillus (Actinomyces). Liver manifestation is rare and, in patients with a history of cancer, differential diagnosis with secondary malignant disease can be difficult. Microbiological result is necessary for a correct diagnosis, though not always possible in preoperative workout. The authors present a case of hepatic actinomycosis that mimicked oncological disease and led to a more aggressive surgical approach.

## Introduction

Actinomycosis is a rare chronic granulomatous infection caused by a type of Gram-positive bacillus, Actinomyces. There are 13 different species described, six of them are pathologic [1] and *Actinomyces israelii* is the most prevalent [2]. Cervicofacial region is the most affected area, mainly related to trauma and dental procedures [3]. Abdominal presentation occurs in 20% of patients and is frequently located in the ileocecal region including the appendix [1]. Hepatic involvement is observed in 15% of abdominal infections, only 5% is exclusively located in the liver [4].

Risk factors for hepatic actinomycosis include dental abscesses or caries, previous surgeries, alcohol abuse, diabetes, and hepatic lithiasis. This disease is more frequent in males in their fifth decade of life [4]. We describe a rare case of hepatic actinomycosis presented as a hepatic tumor, mimicking a secondary malignant lesion, in a patient that underwent a previous surgery for ampullary malignant tumor.

## Case presentation

A 52-year-old male with a history of high blood pressure, smoking, and moderate alcohol habits, was hospitalized for asymptomatic obstructive jaundice. Endoscopic retrograde cholangiopancreatography showed an ampullary tumor and biopsies revealed a high-grade dysplasia adenoma. After a multidisciplinary team decision, the patient underwent pyloric preservation pancreaticoduodenectomy. No events were registered and the patient was discharged on 10th postoperative day.

Tumor was classified as poorly differentiated adenocarcinoma of the biliopancreatic confluence with lymph node metastasis - pT1G2N1 (according to tumors, nodes, and metastases {TMN} classification [5]). Chemo and radiotherapy were proposed as adjuvant therapy. After systemic treatment, the patient was monitored with computerized tomography (CT) and tumor blood markers every four months for the first two years and thereafter every six months.

**Figure 1:**
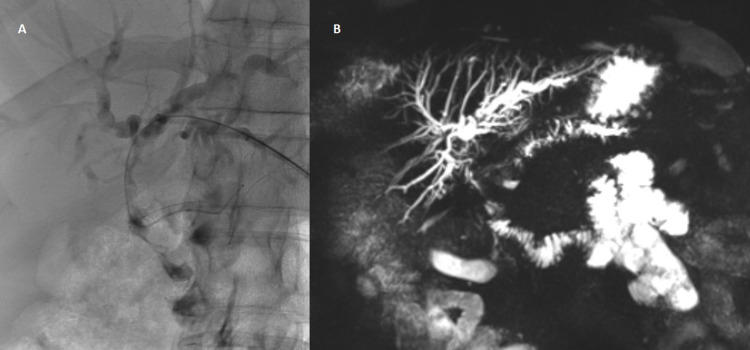
Cholangiography (A) and cholangio-MRI (B) showing dilatation of intrahepatic bile ducts.

Four years after the index surgery, patient was admitted to the hospital for cholangitis. Imaging study showed dilation of intrahepatic bile ducts, mostly on the left side, without a visible mass (Figures 1A, 1B). The tumor markers were normal. Secondary to this stenosis of hepaticojejunal anastomosis, patient had multiple episodes of cholangitis, some of which required hospitalization for intravenous antibiotics, with maximum total bilirubin of 13 mg/dL. After two consecutive attempts of percutaneous treatment with balloon dilation, surgical revision of anastomosis was performed - resection of the stenosis and creation of a new hepaticojejunostomy. The patient was discharged on day seven, without complications.

One year later, during follow-up, an increase of tumor markers (carbohydrate antigen {CA 19-9} of 1142 U/mL and carcinoembryonic antigen {CEA} of 6.46 ng/mL) was detected. Alpha-fetoprotein was not measured. The remaining analytical study was normal, leukocytes of 5.4x10^9^/L with 67.1% of neutrophils, hemoglobin was 13.6 g/dL, oxaloacetic transaminase was 32 U/L, alkaline phosphatase was 339 U/L, international normalized ratio (INR) was 1.2, and total bilirubin was 0.65 mg/dL.

**Figure 2:**
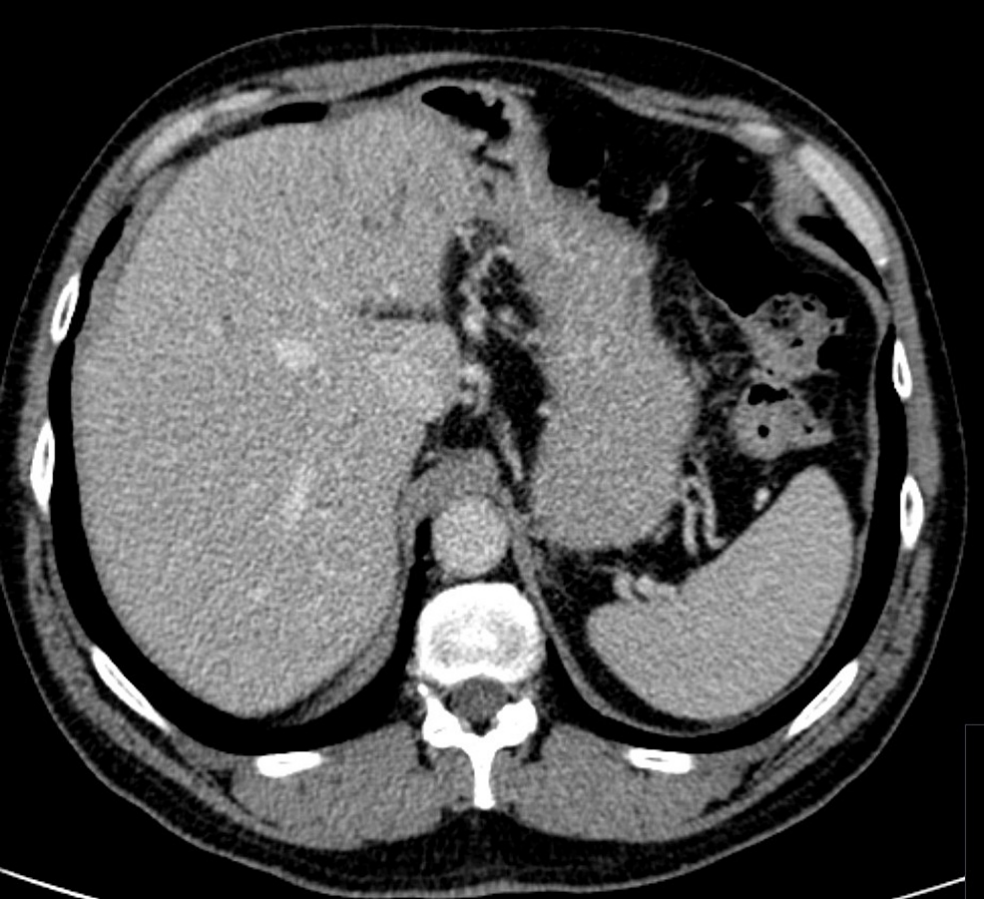
Normal CT scan of the abdomen.

**Figure 3:**
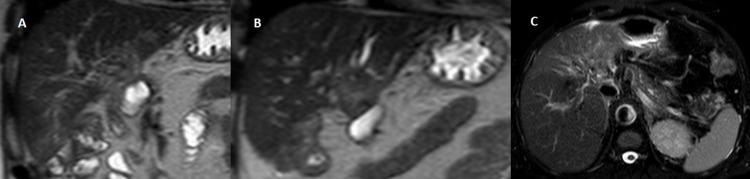
MRI shows a slightly hyperintense T2w irregular lesion in lateral segments of left lobe, with dilatation of the intrahepatic bile ducts ({A and B} coronal T2w SSh, {C} axial T2w FSE fat sat). T2w SSh: T2-weighted single shot; T2w FSE fat sat: T2-weighted fast spin-echo fat-saturated

**Figure 4:**
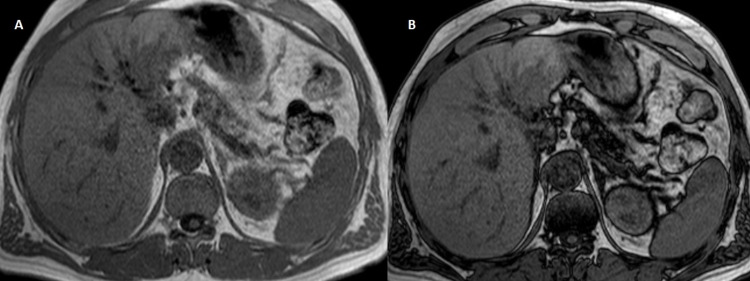
MRI shows a hypointense in T1w without signal loss in opposed-phase ({A} axial T1w GRE phase and {B} axial T1w GRE op-phase). T1w GRE: T1-weighted gradient-echo; T1w GRE op-phase: T1-weighted gradient-echo opposed-phase

**Figure 5:**
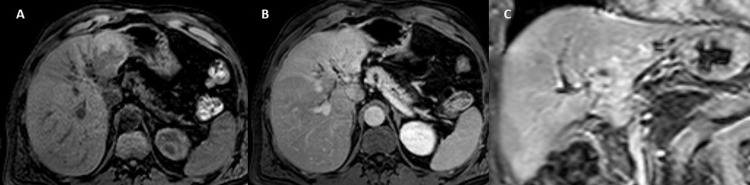
MRI shows heterogeneous hyperenhancement after extracellular contrast ({A} axial T1w pre, {B} axial T1w post-Gd portal phase, and {C} coronal T1w post-Gd delayed phase). T1w post-Gd: T1-weighted post-gadolinium

**Figure 6:**
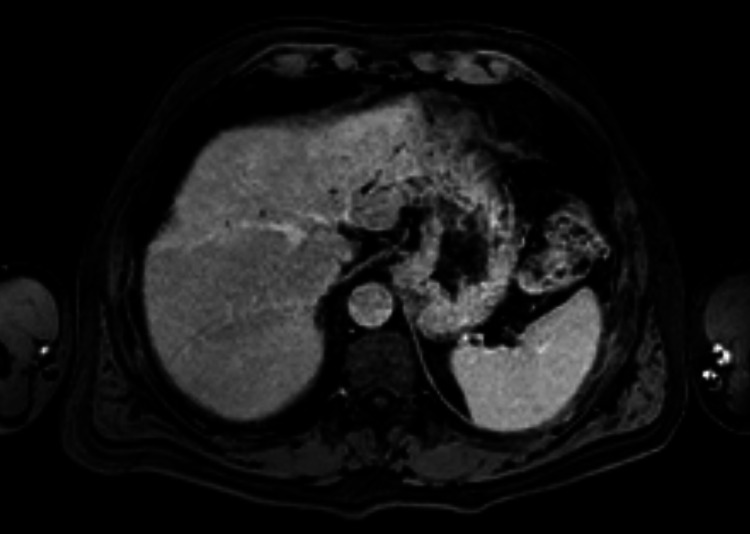
MRI shows in the cranial planes the lesion abuts the left suprahepatic vein (axial T1w post-Gd delayed phase). T1w post-Gd: T1-weighted post-gadolinium

**Figure 7:**
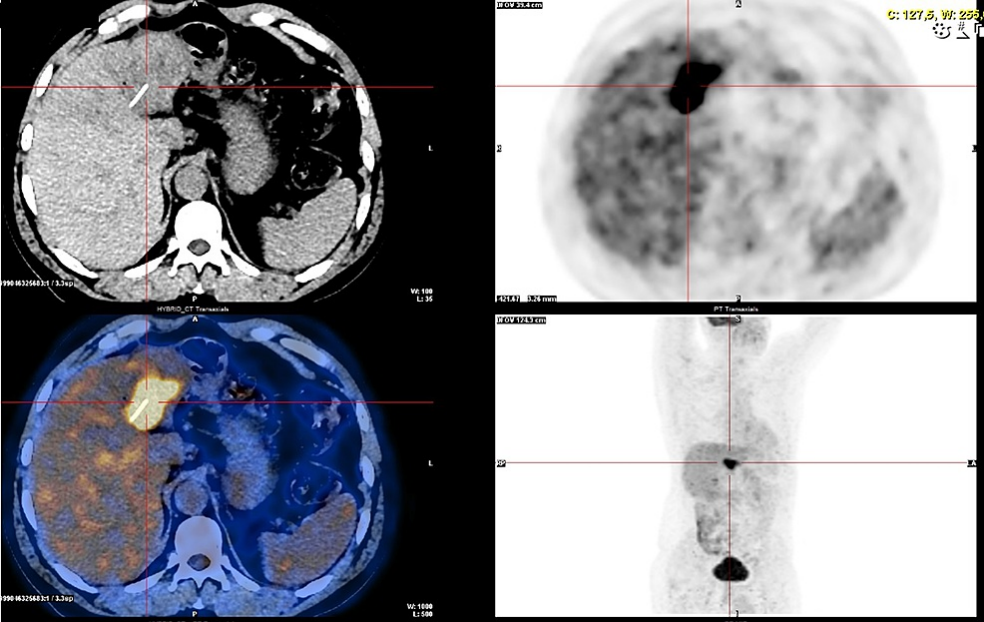
PET-CT consistent with secondary lesion. PET-CT: positron emission tomography-computed tomography

Clinically, the patient was asymptomatic, without constitutional symptoms, anicteric, and with a normal physical examination. Abdominal CT was normal (Figure 2). Abdominal magnetic resonance image (MRI) showed a slightly hyperintense T2-weighted (T2w) irregular lesion, measuring 42x29 mm, in lateral segments of left lobe, with dilation of intrahepatic bile ducts (Figures 3A-3C), hypointense in T1-weighted (T1w) without signal loss in opposed-phase (Figures 4A, 4B) with heterogeneous hyperenhancement after extracellular contrast (Figures 5A-5C). In cranial planes, the lesion abuts the left suprahepatic vein (Figure 6). These findings were highly suggestive of malignant disease. Positron emission tomography-computed tomography (PET-CT) was consistent with secondary lesion (standard uptake value {SUV} of 12.73) (Figure 7). Upper and lower endoscopy were normal.

Multidisciplinary oncologic team decided for a surgical approach. The patient was submitted to an uneventful left hepatectomy. The patient was discharged on day nine. Histologic analysis revealed hepatic abscess compatible with actinomycosis. The patient was treated with doxycycline for six weeks. After the treatment tumor markers normalized and the patient was asymptomatic. The patient was kept under surveillance with CT and tumor markers every six months for two years.

## Discussion

Isolated hepatic actinomycosis is a rare condition. Fever, weight loss, abdominal pain, and hepatic cystic lesion may suggest this entity as a differential diagnosis, although unspecific symptoms usually lead to late diagnosis [6]. Microbiological confirmation from actinomycotic abscess content is necessary [7]. The exclusion of malignancy is one of the main purposes of investigation, especially when patients present with a single liver mass associated with an increase of tumor markers [2]. Some studies show a correlation with increased levels of CA 19-9, but with values ​​lower than those found in malignant disease [1].

In the current case, etiological doubt was based on the past oncological history, namely, a metastasis from the carcinoma excised years ago. In fact, it is described that abdominal actinomycosis can resemble a malignant disease presenting with abdominal pain, asthenia, and weight loss [6]. Less than 10% of cases are diagnosed preoperatively [2]. Despite hematogenic route remains the most frequent, association with direct spread from gallbladder infection, peptic ulcer perforation, biliary and pancreatic stents, or its manipulation is described [7].

In up to 66% of cases, hepatic actinomycosis presents as a single hypodense lesion, as in this patient. More than 40% of imaging examinations may not distinguish from a malignant lesion; however, hepatic MRI typically shows an image characterized by hypointensity in T1 and hyperintensity in T2, as happened in this case. There are no specific findings in CT scan and ultrasonography, and positive blood cultures are extremely difficult to obtain. Tumor marker CA 19-9 might be increased, though levels are usually lower than seen in malignant disease [1].

In this case, we were treating a young patient with a single, resectable liver lesion, with no evidence of disseminated disease that appears five years after resection of an ampullary tumor. The hypothesis of liver metastasis seemed less likely considering the elapsed time since the first surgery. Metastases from a digestive primary neoplasm were unlikely with a normal endoscopic study. The hypothesis of other primary liver tumors such as hepatocellular carcinoma was unlikely given the lesions’ imaging characteristics and the absence of liver cirrhosis [8]. The findings on MRI, suspected of malignant disease, raised the possibility that it was an intrahepatic cholangiocarcinoma, despite having unusual imaging features. Biopsy was not considered since the lesion was resectable and the results of the biopsy wouldn’t change the treatment plan.

At this moment, there are no surgical treatment guidelines for hepatic actinomycosis [1]. Treatment options are still in debate but abscess drainage or surgical debridement and long-term antibiotic therapy are often needed. Intravenous antibiotics and posterior oral switch with no drainage is also an option, after pathogen confirmation through percutaneous biopsy.

This differential diagnosis cannot be neglected since, with the exception of the microbiological examination, there is no proper diagnostic tool for actinomycosis [3]. There aren’t any specific radiological, hematological, or endoscopic findings for this disease [2]. Thus, this present case intends to recall this entity in order to consider it in differential diagnoses, particularly in immunocompetent male patients, with abdominal surgical history or who had previous manipulation of biliary tract.

In this case, medical history was highly suggestive of a secondary lesion, so no preoperative biopsy was attempted, even though several infectious occurrences one year before should have raised the hypothesis of an infectious pathology in the differential diagnosis. Lack of symptomatology, high levels of tumor markers, doubtful MRI, and a positive PET scan deviated the suspicion towards malignancy leading to a more invasive approach.

## Conclusions

Hepatic actinomycosis is a rare entity characterized by indolent evolution and unspecific clinical and imaging features. Differential diagnoses include other liver infectious conditions, as well as malignant tumors (liver metastasis, hepatocellular carcinoma, intrahepatic cholangiocarcinoma, etc.). High level of suspicion is needed to adequate the best treatment option.
